# Comparison of Neoadjuvant Intraarterial Chemotherapy Versus Concurrent Chemoradiotherapy in Patients With Stage IIIB Uterine Cervical Cancer

**DOI:** 10.4021/wjon720w

**Published:** 2014-01-16

**Authors:** Ryuji Kawaguchi, Haruki Nakamura, Sachiko Morioka, Huminori Ito, Yasuhito Tanase, Shoji Haruta, Seiji Kanayama, Shozo Yosida, Naoto Furukawa, Hidekazu Oi, Hiroshi Kobayashi

**Affiliations:** a Department of Obstetrics and Gynecology, Nara Medical University, Nara, Japan

**Keywords:** Cervical cancer, Neoadjuvant intraarterial chemotherapy, Concurrent chemoradiotherapy, Survival, Recurrence

## Abstract

**Background:**

The purpose of this study was to compare the long-term survival of patients with stage IIIB squamous cell carcinoma of the cervix treated with neoadjuvant intraarterial chemotherapy (IA-NAC) versus those treated with concurrent chemoradiotherapy (CCRT).

**Methods:**

We retrospectively reviewed the clinical records of 38 patients with stage IIIB squamous cell carcinoma of the cervix admitted between January 1994 and December 1999 who received IA-NAC followed by abdominal radical hysterectomy (ARH) or radiotherapy (RT). IA-NAC consisted of bilateral infusion via the internal iliac artery of cisplatin, bleomycin and pirarubicin for 2-3 courses. A historical control group of 64 patients who underwent primary CCRT from January 2000 to September 2007 was used for comparison.

**Results:**

In the IA-NAC group, 12 patients (31.6%) with operable tumors underwent ARH, and the remaining 26 patients (68.4%) received RT. The response rates were 86.8% (12 complete response + 21 partial response) for IA-NAC and 98.4% (26 complete response + 37 partial response) for CCRT (P = 0.077), respectively. The 5-year overall survival and disease-free survival rates were 62.4 and 44.5% for IA-NAC and 51.1 and 46.9% for CCRT (P = 0.247 and 0.776), respectively. The 5-year overall survival and disease-free survival rates were 75.0 and 58.3% for the patients receiving IA-NAC followed by ARH, and 55.3 and 37.6% for the patients receiving IA-NAC followed by RT (P = 0.368 and 0.262), respectively.

**Conclusions:**

In the present study, IA-NAC followed by ARH or RT and primary CCRT showed similar survival rates for stage IIIB squamous cell carcinoma of the cervix.

## Introduction

Concurrent chemoradiotherapy (CCRT) is the main treatment for locally advanced cervical cancer [1-3]. Neoadjuvant chemotherapy (NAC) was widely employed until CCRT became the standard, and favorable results have been reported [4-6]. However, the efficacy of NAC has not been confirmed by some researchers [7-11], and its value therefore remains unclear. Neoadjuvant intraarterial chemotherapy (IA-NAC) is another method for delivering NAC as an alternative to systemic chemotherapy. IA-NAC has been reported to achieve beneficial results that cannot be obtained by systemic chemo­therapy or CCRT [12-15], but conclusive evidence is limited. We have employed IA-NAC for treatment of advanced cervical cancer since 1994. In the present study, we compared the therapeutic efficacy of IA-NAC followed by abdominal radical hysterectomy (ARH) or radiotherapy (RT) with CCRT for stage IIIB cervical cancer.

## Materials and Methods

After obtaining Institutional Review Board approval, we retrospectively reviewed the medical records of patients treated for stage IIIB cervical cancer at Nara Medical University Hospital between January 1994 and September 2007.

### Patients

A total of 102 patients with stage IIIB primary cervical cancer treated between January 1994 and September 2007 were evaluated in this study. All patients had primary, previously untreated, histologically confirmed invasive squamous cell carcinoma. No patients were pregnant and all had adequate bone marrow, renal and hepatic function.

The patients were staged based on the FIGO clinical staging criteria for cervical cancer. The clinical staging was determined by pelvic examination, colposcopy with biopsy, chest X-ray, cystoscopy, sigmoidoscopy and intravenous pyelography. Tumor size was defined as the longest diameter determined by clinical staging performed by gynecologic oncologists. Magnetic resonance imaging (MRI) and computed tomography (CT) were performed on all patients. The following parameters were obtained from the imaging studies: tumor size; parametrial invasion; lymph node metastasis; and vaginal involvement. Patients with para-aortic lymph node enlargement were excluded.

### Treatment plan

The following two treatments were employed in chronological order. Between January 1994 and December 1999, 38 patients received IA-NAC followed by ARH or RT. Briefly, after receiving IA-NAC, the patients were clinically reassessed and classified as suitable or unsuitable for ARH. The latter patients were treated with RT. Postoperative RT was given to all patients. Subsequently, between January 2000 and September 2007, 64 patients received primary CCRT.

### IA-NAC

Under local anesthesia, the Seldinger technique was used to place bilateral polyethylene catheters of 5-French diameter in the internal iliac artery just distal to the superior gluteal artery. Pelvic angiography was performed during catheterization to ensure correct positioning of the catheter and effective tumor perfusion. After each treatment, the catheters were removed and sandbags were used to apply firm pressure over each groin area for 6 h. The regimen for day 1 involved administration of 20 mg of bleomycin and 40 mg of epirubicin within 30 min, followed by infusion of 100 mg of cisplatin over 2 h with vigorous hydration. All drugs were administered in divided doses via the bilateral internal iliac artery. IA-NAC was administered for 2-3 courses every 3 weeks. The number of courses given depended on the tumor response and the operability was determined by MRI and pelvic examination.

### ARH

Following IA-NAC, a type III ARH with pelvic lymphadenectomy was performed in patients with stage IIIB disease responding to IA-NAC, if possible. After surgery, the patients received adjuvant systemic RT.

### RT

The patients were treated with anteroposterior and posteroanterior parallel-opposed ports of external beam radiotherapy (EBRT). The dose of EBRT was 50 Gy delivered in 25 fractions. Center shields (4-cm width at the midline) were set up after delivery of 40 Gy. The radiation field included the primary tumor, uterus, paracervical, parametrial and uterosacral regions, and pelvic lymph nodes. High-dose rate intracavitary RT was delivered once per week with a fraction dose of 6 Gy at point A three or four times.

### CCRT

Since 2000, weekly cisplatin in conjunction with RT has been used as the preferred primary treatment. The weekly cisplatin regimen started with a dose of 40 mg/m^2^ on day 1 of external RT, at 1 - 4 h before RT initiation. The Eastern Cooperative Oncology Group (ECOG) toxicity criteria were used for monitoring and documentation of hematologic toxicities. Chemotherapy was delayed for an ANC of less than 500/mm^3^, or a platelet count of less than 50,000/mm^3^. We injected granulocyte colony-stimulating factor and intravenous antimicrobial agents only when the patient had febrile neutropenia, defined as an oral temperature above 38.5 °C and persistent neutropenia with serious complications such as pneumonia or any type of progressive infection.

### Response assessment

The tumor dimensions were measured by MRI before and after therapy. Patients were evaluated for their tumor response according to the Response Evaluation Criteria in Solid Tumors (RECIST).

### Follow-up

A gynecologist followed the patients for 1 month after treatment. Subsequently, the follow-up was every 3 months for the first 2 years, and every 3 - 6 months thereafter. The follow-up intervals varied for patients suspected of having recurrent disease, based on the individual situations. Patients with suspicious symptoms, such as signs at physical examination during the follow-up period, were given additional tests (histologic examination, abdominopelvic CT, pelvic MRI, and so on) to confirm the presence of recurrent disease.

### Statistical analysis

Statistical analyses were performed using Statistical Package for Social Sciences (SPSS) 11.0 software (SPSS Inc., Chicago, IL, USA). The unpaired Student’s t-test for continuous variables and either the chi-square or Fisher’s exact test for categorical data were used to evaluate the statistical significance of differences in the clinical characteristics between the two groups. For all statistical tests, the level of significance was set at P < 0.05. Survival analyses were performed by the Kaplan-Meier method, and statistical significance was determined by the log-rank test. Overall survival (OS) was calculated by evaluating the time from the end of treatment to the last follow-up or death. Disease-free survival (DFS) was evaluated by calculating the time from the end of treatment to any disease progression (local recurrence or distant metastasis).

## Results

A total of 102 patients were evaluated in this study, comprising 38 patients in the IA-NAC group and 64 patients in the CCRT group. The patient characteristics and associations are shown in Table 1. There were no significant differences in age at diagnosis, performance status, pelvic lymph node status and pretreatment level of squamous cell carcinoma antigen between the two groups. The tumor size was larger in the IA-NAC group, and the difference was significant (P = 0.012).

**Table 1:** Clinical Characteristics of All the Patients With Stage IIIB Cervical Cancer

	IA-NAC group (n = 38)	CCRT group (n = 38)	P value
Age (years)			
Mean ± SD	56.2 ± 9.0	58.9 ± 12.4	0.612
ECOG performance status (n, %)			
0	37 (97.4%)	60 (93.5%)	0.857
1-2	1 (2.6%)	4 (6.5%)	
Tumor dimension (mm)			
Mean ± SD	55.6 ± 16.7	47.5 ± 14.9	0.012
Pelvic lymph node status (n, %)			
Negative	23 (60.5%)	54 (84.4%)	0.071
Positive	15 (39.5%)	11 (17.2%)	
Serum SCC (ng/ML)			
Mean ± SD	25.1 ± 33.0	24.1 ± 38.8	0.893

**Figure 1:**
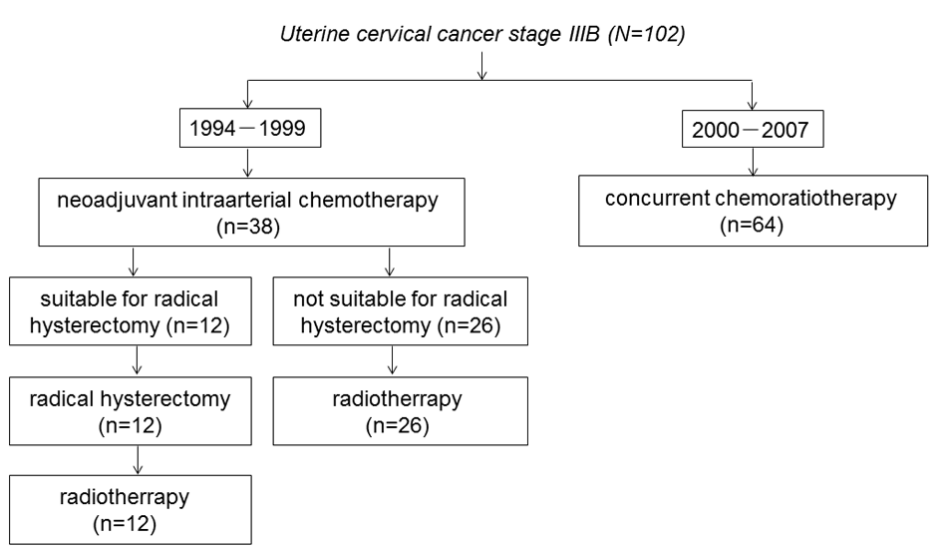
The trial profile of the flow of patients.

Figure 1 shows the trial profile of the flow of patients in the study and their last follow-up status. Between January 1994 and December 1999, 38 patients received IA-NAC. Twelve patients were judged to be suitable for ARH, and subsequently received RT. The remaining 26 patients were judged to be inoperable, and received RT. Between January 2000 and September 2007, 64 patients received CCRT. The baseline characteristics of the patients in the IA-NAC group showed no significant differences between the ARH group and the RT group (Table 2).

**Table 2:** Clinical Characteristics Patients With Treated With IA-NAC

	ARH group (n = 12)	RT group (n = 26)	P value
Age (years)			
Mean ± SD	53.7 ± 8.6	57.3 ± 9.1	0.246
ECOG performance status (n, %)			
0	12 (100%)	26 (93.5%)	0.857
1-2	0 (0%)	1(6.5%)	
Tumor dimension (mm)			
Mean ± SD	53.8 ± 1637	56.4 ± 17.2	0.659
Pelvic lymph node status (n, %)			
Negative	7 (58.3%)	16 (61.5%)	0.583
Positive	5 (41.7%)	10 (38.5%)	
Serum SCC (ng/ML)			
Mean ± SD	10.9 ± 10.2	31.6 ± 37.7	0.073

All 102 patients were evaluated for their response to treatment. In the IA-NAC group, clinical responses (complete response (CR) + partial response (PR)) occurred in 33 of 38 patients (86.8%), comprising CR in 15 patients (39.4%) and PR in 18 patients (47.4%), with stable disease (SD) in the remaining five patients (13.2%) (Table 3). In the CCRT group, clinical responses occurred in 63 of 64 patients (98.4%), comprising CR in 26 patients (40.6%) and PR in 37 patients (57.8%), with SD in the remaining one patient (1.6%) (Table 3). There were no significant differences in the local response between the IA-NAC group and the CCRT group (P = 0.343).

**Table 3:** Response to IA-NAC or CCRT

	IA-NAC group (n = 38)		CCRT group (n = 64)	
	No. of patients	%	No. of patients	%
Complete response	15	39.4	26	40.6
Partial response	18	47.4	37	57.8
Stable disease	5	13.2	1	1.6
Response rate	33	86.8	63	98.4

The patterns of failure and death are shown in Table 4. Twenty patients (52.6%) in the IA-NAC group and 33 patients (51.6%) in the CCRT group relapsed. Among the 20 patients with disease recurrence in the IA-NAC group, nine (23.7%) had local failure, nine (23.7%) had distant failure and two (5.2%) had combined failure. The median time to relapse was 41.1 months (range, 4.8 - 48.3 months). Among the 33 patients with disease recurrence in the CCRT group, 22 (34.3%) had local failure, six (9.3%) had distant failure and two (3.1%) had combined failure. The median time to relapse was 27.9 months (range, 3.5 - 28.8 months). Regarding the incidence of lesion relapse, the relapse of distant lesions in the patients who received IA-NAC followed by ARH or RT (23.7%) was significantly higher than that for patients who received CCRT (9.3%) (P = 0.048).

**Table 4:** The Patterns of Failure and Death

Outcome	IA-NAC group (n = 38)		CCRT group (n = 64)	
	No. of patients	%	No. of patients	%
Progression status				
Relapse	20		33	
Local	9	23.7	22	34.3
Distant	9	23.7	6	9.3
Combined	2	5.2	2	3.1
No evidence of disease	18	47.4	31	48.3
				
Survival status				
Dead	17	44.7	34	53.1
Alive	21	55.3	30	46.9

**Figure 2:**
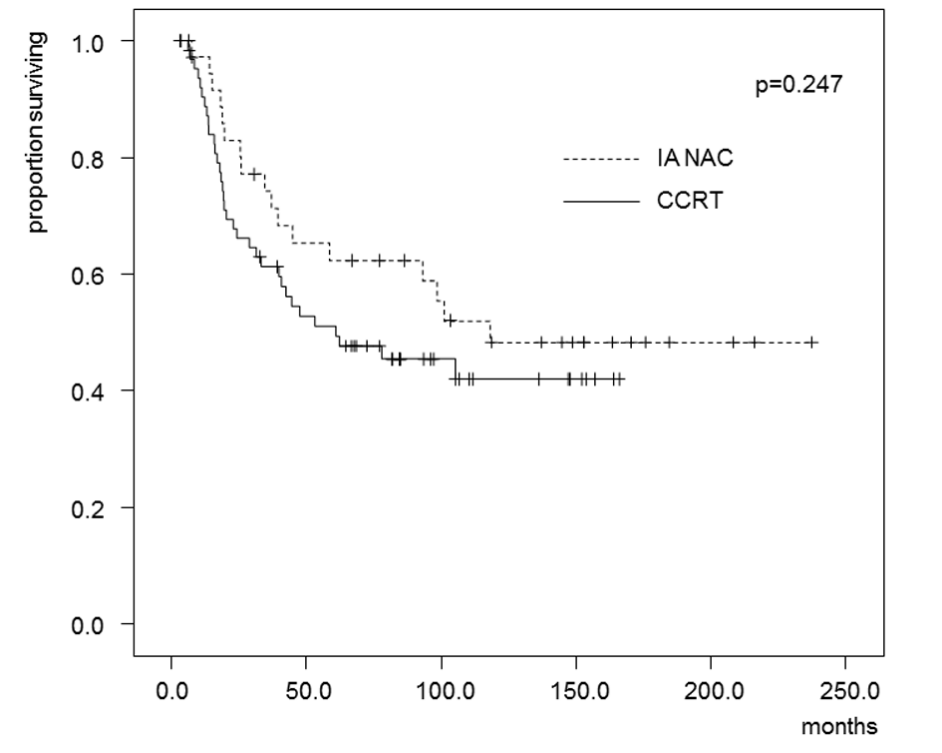
Kaplan-Maier estimates of overall survival for patients who received IA-NAC or CCRT. The 5-year survival rate was 62.4% with IA-NAC and 51.1% with CCRT.

**Figure 3:**
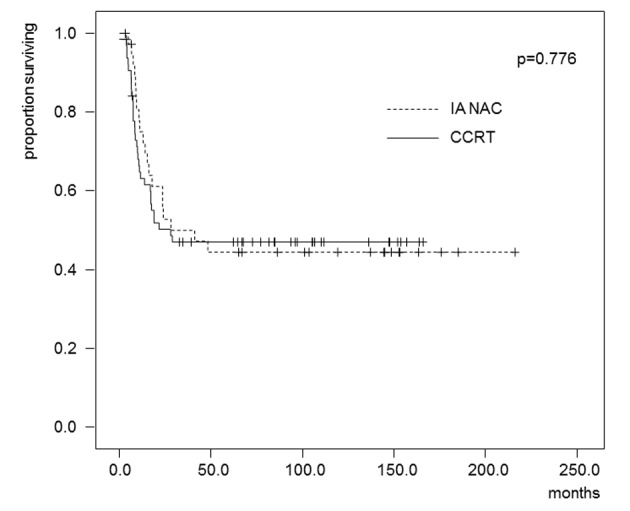
Kaplan-Maier estimates of disease-free survival for patients who received IA-NAC or CCRT. The 5-year survival rate was 44.5% with IA-NAC and 46.9% with CCRT.

The estimated OS and DFS rates are shown in Figures 2 and 3. The 5-year OS rates were 62.4% in the IA-NAC group and 51.1% in the CCRT group, with no significant difference (P = 0.247). The 5-year DFS rates were 44.5% in the IA-NAC group and 46.9% in the CCRT group, also with no significant difference (P = 0.776).

**Figure 4:**
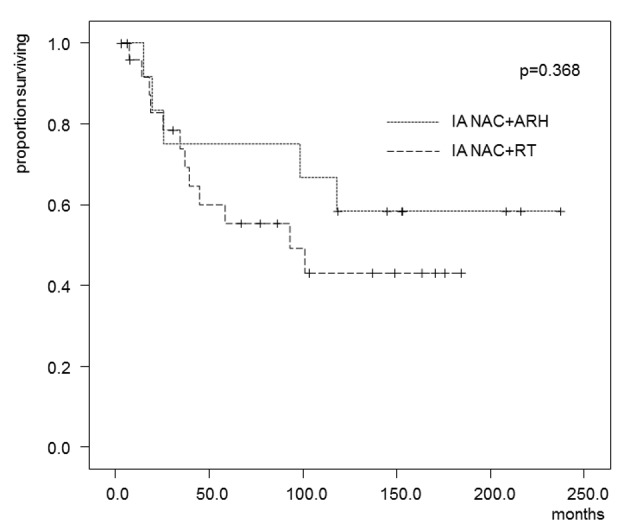
Kaplan-Maier estimates of overall survival for patients who received IA-NAC + ARH or IA-NAC + RT. The 5-year survival rate was 75.0% with IA-NAC and 55.3% with CCRT.

**Figure 5:**
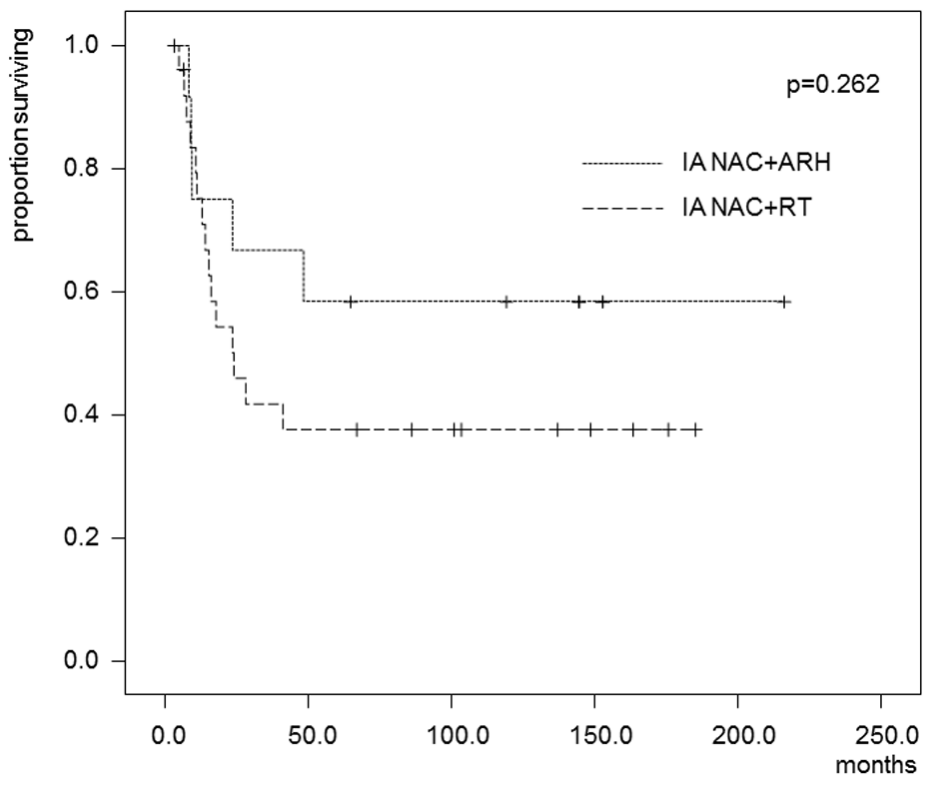
Kaplan-Maier estimates of disease-free survival for patients who received IA-NAC + ARH or IA-NAC + RT. The 5-year survival rate was 58.3% with IA-NAC and 37.6% with CCRT.

In the patients who received IA-NAC, the 5-year OS rates were 75.0% in the ARH group and 55.3% in the RT group (Fig. 4), with no significant difference (0.368). The 5-year DFS rates were 58.3% in the ARH group and 37.6% in the RT group (Fig. 5), also with no significant difference (P = 0.262).

## Discussion

There are theoretical advantages for using NAC prior to definitive treatment by surgery or RT. NAC may promote the efficiency of definitive treatment for local control by adequately down-staging the tumor before the intratumoral blood flow is affected by the definitive treatment. In particular, IA-NAC can increase the tumor exposure to high drug concentrations, while decreasing the drug delivery to systemic tissues.

In the gynecologic field, the most progressive research concerning the efficacy of NAC has been conducted in advanced cervical cancer. Effectiveness of a briefer, but more intensive, neoadjuvant treatment for locally advanced cervical cancer can be expected. In patients with stage IIIB cervical cancer, even systemic administration of chemotherapeutic drugs has not been successful because the chemotherapy response rates decrease as the tumor size increases [16]. Therefore, IA-NAC could offer the therapeutic advantages of providing increased drug concentrations at the tumor level and lower toxicity if a first-pass effect can be achieved.

Cisplatin has been the most effective drug for patients with cervical cancer [17]. Scarabelli et al [13] reported an extremely high response rate (91.7%) in 36 patients with stage IIIB-IVA cervical cancer treated with bilateral infusion via the iliac artery of bleomycin, doxorubicin and cisplatin. The major toxicities were hematologic (19.4%, grade 3-4), renal (2.8%, grade 2) and gastrointestinal (19.4%, grade 1-2). These results suggested that the regimen was effective and feasible. Therefore, we chose cisplatin, bleomycin and epirubicin. We performed intraarterial infusion through the internal iliac artery, consistent with the route used by Scarabelli et al [13].

Although several studies have provided evidence for the effectiveness of NAC in locally advanced cervical cancer, new strategies combining chemotherapy and conventional therapy remain controversial. One reason may be the appropriate stages for stage comparisons. Our study focused on patients with stage IIIB cervical cancer, an inoperable stage in which RT or CCRT has been the worldwide standard. The effects of IA-NAC on the response rate, operability and survival rate were investigated. We have also reviewed studies involving treatment with IA-NAC for stage IIIB squamous cell carcinoma of the uterine cervix [18-24]. The regimens were varied. A median response rate of 80% (range: 69.2-100%) and infrequent CR rates have been reported for combination therapy with a platinum agent, and an operability rate similar to the response rate has been reported in several studies. Our study had a response rate of 86.8% and an operability rate of 37.2%.

Until 1998, the standard primary therapy for patients with FIGO stage IIB-IVA cervical cancer consisted of RT without chemotherapy. From 1999 to date, the standard primary treatment has been CCRT [1-3, 25, 26]. CCRT with cisplatin reduces the relative risk of death from cervical cancer by 30-50% by decreasing the disease recurrence. CCRT has shown significant benefits for local recurrence and may be of benefit for distant recurrence. However, more than 50% of patients with recurrence were found to have distant metastases following CCRT.

NAC followed by definitive surgery has been studied in comparison with surgery alone or RT alone. However, few studies have compared NAC followed by definitive surgery with CCRT. One retrospective study of NAC followed by surgery versus CCRT in patients with IB2-IIIB cervical cancer failed to demonstrate a significant improvement in survival [27].

In this paper, we have presented the results for a series of 38 patients with a primary diagnosis of uterine cervical cancer stage IIIB treated with a platinum-based regimen as IA-NAC followed by ARH and/or RT compared with 64 patients treated with CCRT. The effects of IA-NAC on patient outcomes have not been adequately defined in patients with uterine cervical cancer stage IIIB. In the present study, despite a good tumor response rate (86.8%) for IA-NAC, only 31.6% of patients who underwent IA-NAC were judged to be operable. The 5-year OS rates were 62.4% in the IA-NAC group and 51.1% in the CCRT group, but the difference was not significant (P = 0.247). A probable explanation for the poor OS despite the satisfactory rate of initial response may be enhancement of accelerated tumor proliferation during treatment. The tumor masses would still be regressing while the subclinical clonogenic cell repopulation is accelerating, and the gain would eventually result in the development of a relapse. Therefore, in patients with locally advanced cancer of the cervix, improvements in local control will not translate into improved long-term outcomes.

IA-NAC was effective in reducing the tumor volume, increasing the clinical response rate, improving the operability in patients with stage IIIB cervical cancer and improving the prognosis of patients with locally advanced cervical cancer. However, no reports have noted an improvement in the prognosis of patients with stage IIIB cervical cancer who did not respond to NAC [5, 12, 13, 19]. In the present study, there was no difference in the prognosis between the patients who responded to IA-NAC and those who did not.

We tried IA-NAC for stage IIIB cervical cancer and compared it with CCRT. Our study showed that the long-term survival rates did not differ significantly between patients with stage IIIB cervical cancer treated with IA-NAC and CCRT. In other words, IA-NAC did not lead to an improvement in the survival rates compared with those treated with CCRT in our institution. However, the number of patients treated was small, and therefore prospective randomized long-term follow-up trials comparing IA-NAC followed by ARH and/or RT with CCRT will be necessary to establish the role of IA-NAC in the treatment of patients with stage IIIB cervical cancer.
